# Sperm Adhesion Molecule 1 (SPAM1) Distribution in Selected Human Sperm by Hyaluronic Acid Test

**DOI:** 10.3390/biomedicines10102553

**Published:** 2022-10-13

**Authors:** María José Gómez-Torres, Paula Sáez-Espinosa, Paula Manzano-Santiago, Laura Robles-Gómez, Natalia Huerta-Retamal, Jon Aizpurua

**Affiliations:** 1 Departamento de Biotecnología, Universidad de Alicante, 03690 Alicante, Spain; 2 Cátedra Human Fertility, Universidad de Alicante, 03690 Alicante, Spain; 3 IVF Spain, Reproductive Medicine, 03540 Alicante, Spain

**Keywords:** hyaluronic acid, hyaluronidase PH-20, male infertility, spermatozoa, sperm capacitation

## Abstract

The failures of binding to the oocyte zona pellucida are commonly attributed to defects in the sperm recognition, adhesion, and fusion molecules. SPAM1 (sperm adhesion molecule 1) is a hyaluronidase implicated in the dispersion of the cumulus-oocyte matrix. Therefore, the aim of this study was to characterize the SPAM1 distribution in the different physiological conditions of human sperm. Specifically, we evaluated the location of the SPAM1 protein in human sperm before capacitation, at one and four hours of capacitation and after hyaluronic acid (HA) selection test by fluorescence microscopy. Sperm bound to HA were considered mature and those that crossed it immature. Our results detected three SPAM1 fluorescent patterns: label throughout the head (P1), equatorial segment with acrosomal faith label (P2), and postacrosomal label (P3). The data obtained after recovering the mature sperm by the HA selection significantly (*p* < 0.05) highlighted the P1 in both capacitation times, being 79.74 and 81.48% after one hour and four hours, respectively. Thus, the HA test identified that human sperm require the presence of SPAM1 throughout the sperm head (P1) to properly contact the cumulus-oocyte matrix. Overall, our results provide novel insights into the physiological basis of sperm capacitation and could contribute to the improvement of selection techniques.

## 1. Introduction

Fertilization is a biological event that entails highly synchronized interactions between the spermatozoon and the oocyte. It is well known that the sperm acquire the capacity to fertilize the oocyte during their residence in the female reproductive tract, a process called sperm capacitation [1]. Capacitation is distinguished by complex structural and physiological sperm modifications, such as membrane cholesterol depletion, glycoconjugate relocation, protein residue phosphorylation, and hyperactivation [2,3]. To complete fertilization, the sperm must release the acrosomal hydrolytic enzymes contained in the acrosome to cross the zona pellucida and fuse with the oocyte membrane [4].

In previous research, the role of the chaperone HSPA2 (heat shock protein 2) in the reorientation of human sperm receptors involved in zona pellucida recognition has been described [5]. Specifically, the model proposes that HSPA2 mediates the orientation of SPAM1 (sperm adhesion molecule 1) and ARSA (arylsulfatase A) on the sperm surface. This complex is oriented in such a way that SPAM1 is first exposed to disperse the cells of the cumulus that surround the oocyte and as the sperm advance, the complex is reoriented and exposes ARSA, involved in the recognition of the zona pellucida [5,6]. 

Precisely, SPAM1 is a GPI-linked protein that it is widely conserved in all mammalian species [7,8,9,10]. It has been reported that SPAM1 is the major testicular hyaluronidase, being located at the testis [9] and in all regions of the epididymal epithelium [10,11]. Moreover, it is known that the SPAM1 secreted form presents an intact GPI anchor and can be detected in soluble and insoluble (epididymosomes) fractions of the epididymal luminal fluid [9,12]. Among the different events in which SPAM1 participates during fertilization [7,10,13,14], it is worth highlighting its involvement in cumulus oophorous complex (COC) dispersion, due to the insoluble hyaluronidase activity at neutral pH [15]. The COC encircles the oocyte and involves the cumulus cells and their extracellular matrix. It should be noted that, during natural fertilization, only the sperm that pass through the extracellular matrix can penetrate the zona pellucida and fertilize the oocyte [16]. In particular, the extracellular matrix is a complex structure, whose main component is hyaluronic acid (HA), which is produced by the cells of cumulus after the levels of luteinizing hormone rise [17]. 

The HA acts as a natural sperm selector, since it has been recorded that those sperm that interact with HA have better morphological characteristics, an intact acrosome, and lower DNA fragmentation rates [18,19]. Furthermore, sperm with hyaluronidase activity are more likely to cross the extracellular matrix, bind to the zona pellucida and fertilize the oocyte [20]. The HA binding test is further considered as a sperm biomarker of great interest, since this technique allows the selection of mature sperm during intracytoplasmic sperm injection (ICSI), contributing to a significant improvement in fertilization rates [21] and embryo quality [22,23]. However, its use has raised recent controversy due to the absence of significant differences in the fertilization rate or the quality of the embryo after microinjecting sperm selected by HA test compared to other physiological treatments [24], which highlights the need for further research. 

Hence, in this study, we have addressed a detailed characterization of SPAM1 protein location in human sperm. Especially, we analyzed the localization of this protein before sperm capacitation, at one and four hours of capacitation and after HA selection test by fluorescence microscopy. Additionally, we evaluated acrosome reaction induction and tyrosine phosphorylation as sperm physiological control biomarkers.

## 2. Materials and Methods

### 2.1. Experimental Design

This research was approved by the ethical committee of the University of Alicante according to the Declaration of Helsinki principles. Semen samples were processed to obtain the sperm in different physiological conditions: noncapacitated sperm (NCS), one-hour capacitated sperm (CS1), four-hour capacitated sperm (CS4), mature and immature sperm selected by HA after one-hour capacitation (MS1 and IS1), mature and immature selected by HA after four-hour capacitation (MS4 and IS4). SPAM1 protein was evaluated in all experimental conditions, while acrosome reaction induction and tyrosine phosphorylation were used as control biomarkers in NCS, CS1, and CS4. One-hour capacitation was chosen in concordance with the WHO swim-up protocol [25] and four-hour capacitation based on a previous study [26,27]. 

### 2.2. Semen Samples Analysis

Semen samples were obtained from ten normozoospermic [25] donors through signed informed consent after three to four days of abstinence by masturbation. The samples were allowed to liquefy for 15 min at room temperature and basic seminogram analyses were conducted at the laboratory of the Department of Biotechnology (University of Alicante) following the WHO guidelines [25]. Sperm concentration and motility were assessed using Makler (BioCare Europe, Rome, Italy) counting chamber, morphology by Papanicolaou staining (Panreac Química S.L.U., Barcelona, Spain), and viability was studied using eosin-nigrosine assay (Projectes i Serveis R + D S.L., Paterna, Spain). 

### 2.3. Sperm Capacitation by Swim Up

The seminal plasma was removed by centrifugation for 10 min at 250 g. Then, the pellet was washed with human tubal fluid medium (HTF, Origio, Måløv, Denmark) and divided into three aliquots, one to fix the noncapacitated sperm and the others were destined for one- and four-hour in vitro capacitation. The capacitation was performed by swim-up in HTF medium (Origio) supplemented with 5 mg/mL of bovine serum albumin (BSA, Sigma-Aldrich, Saint Louis, MO, USA) at 37 °C and 5.5% (*v*/*v*) of CO_2_ [25]. Next, supernatant fraction was collected and washed three times in phosphate buffered saline without calcium and magnesium (PBS, Biowest, Nuaillé, France) by centrifugation (250 g, 10 min). Following the capacitation, the concentration, motility, and viability of each sperm recovery were analysed. This methodology was performed following previous protocols of our group [28]. At this point, the recovered motile sperm were divided into three aliquots: one for the HA test, another for the study of the acrosomal state, and the other for fixation assigned to the analysis of tyrosine phosphorylation and SPAM1.

### 2.4. Hyaluronic Acid Sperm Selection

A 15 µL drop of CS1 and CS4 was connected with a pipette tip to a 15 µL drop of SpermSlow medium (Origio) in a Petri dish. Subsequently, the spermatozoa were incubated for 10 min at 37 °C under oil (FertiCult Mineral Oil) following the manufacturer’s instructions. In this way, the sperm with HA receptors, upon encountering the SpermSlow medium, were trapped in the junction area of the two drops and received the name of mature sperm (MS), whereas sperm without these receptors were able to swim through the droplet and were termed immature (IS). This methodology was performed following previous protocols of our group [29].

### 2.5. Induction and Evaluation of Acrosomal Reaction

The induction of the acrosome reaction was performed by 10 µM of calcium ionophore A23187 (Sigma-Aldrich) and 2 mM of calcium chloride (Panreac Química S.L.U, Barcelona, Spain) for one hour at 37 °C and 5.5% (*v*/*v*) CO_2_, following previous protocols of our group (Sáez-Espinosa et al., 2020). Only calcium chloride was added to the controls to assess spontaneous acrosome reaction. 

To assess the acrosomal status, 5 µL of each physiological condition (controls and induced samples) were placed on coverslips and fixed in methanol for 30 min. After the smear was dry, cells were washed three times in PBS and unspecific bindings were blocked using 2% (*w*/*v*) BSA-PBS for 30 min. The smears were then incubated in the dark with Pisum sativum agglutinin lectin conjugated with fluorescein-5-isothiocyanate (PSA-FITC, Sigma-Aldrich) at a concentration of 50 µg/mL for 30 min. After three washes in PBS, samples were mounted using Vectashield and 4′,6-diamidine-2′-phenylindole dihydrochloride (DAPI, Vector Laboratories, Burlingame, CA, USA). DAPI was used to detect the nucleus of the cells and the whole process was conducted at room temperature (Sáez-Espinosa et al., 2020). 

### 2.6. Fixation

All sperm physiological conditions (NCS, CS1, CS4, MS1, IS1, MS4, and IS4) were fixed in paraformaldehyde. The samples were centrifuged, supernatant was discharged, and the pellet was resuspended in 500 µL of 2% (*w*/*v*) paraformaldehyde (Electron Microscopy Sciences, Hatfield, PA, USA) during 45 min at 4 °C. After fixation, paraformaldehyde was replaced with PBS and the samples were kept at 4 °C until their use. 

### 2.7. Immunolocation of Tyrosine Phosphorylation

A total of 5μL of each paraformaldehyde-fixed condition was placed on a coverslip. When smear was dry, cells were washed three times for 5 min with PBS. Smears were then incubated with the primary PY20 anti-phosphotyrosine antibody (1:500) produced in rabbit (Sigma-Aldrich) in blocking solution of 2% PBS-BSA for one hour at room temperature. Subsequently, three washes were made with PBS and the secondary anti-rabbit antibody conjugated with Alexa Fluor 488 (1:100) (Jackson ImmunoResearch, Ely, UK) was added in blocking solution of 2% (*w*/*v*) PBS-BSA for one hour at room temperature. This methodology was performed following previous protocols of our group [30]. Finally, three washes were made with PBS and the assembly was performed with Vectashield with DAPI (Vector Laboratories).

### 2.8. Immunolocation of SPAM1

A total of 5 µL of the paraformaldehyde-fixed samples was deposited on a coverslip. Once cover was dried, samples were rehydrated thrice with PBS and permeabilized with Triton X-100 at 0.2% in PBS for 10 min at room temperature. Coverslips were then washed again with PBS prior to incubation with the primary antibody produced in rabbit anti-SPAM1 (Sigma-Aldrich) diluted in 2% (*w*/*v*) BSA-PBS 1:100 at 4 °C in a humid chamber overnight. After being washed with PBS three times for 5 min, samples were incubated with a polyclonal donkey anti-rabbit IgG-FITC (Thermo Fisher Scientific, MA, USA) antibody in 2% (*w*/*v*) BSA-PBS 1:100 for one hour in darkness. This methodology was performed following previous protocols of our group [31]. Finally, covers were washed in PBS and assembled (Vectashield, Vector Laboratories).

### 2.9. Statistical Analysis

Acrosome reaction, tyrosine phosphorylation, and SPAM1 label were evaluated by Confocal Laser Scanning Zeiss LSM 800 Microscope (Zeiss, Oberkochen, Germany) and Zeiss Imaging Software. Z-stack sections (1040 × 1040 pixels) of the sperm were obtained using an oil 63× objective and 405 nm and 488 nm lasers. Then, the sections were reconstructed using ZEN 2.5 lite software (Zeiss) and at least 200 cells were evaluated for each biomarker. Moreover, the appropriate negative controls performed served to corroborate the specificity of the reagents. Precisely, the PY20 and SPAM1 negative controls were performed omitting the first antibody and for the acrosomal reaction the lectin was omitted.

The Shapiro–Wilk (W) test showed significant sperm biomarker differences in distribution and variance (W = 0.872 to 0.978; *p* < 0.01). The nonparametric Kruskal–Wallis test was used to assess differences between different physiological conditions within each biomarker analyzed. Two-sided *p*-values < 0.05 were statistically significant. All statistical analyses were performed using IBM SPSS Statistics 22.0 (IBM, Armonk, NY, USA).

## 3. Results

### 3.1. Sperm Parameters

All sperm samples involved in this research were normozoospermic according to World Health Organization (WHO) standards [25]. Results of sperm parameters from different physiological conditions: noncapacitated sperm (NCS), one-hour capacitated sperm (CS1), and four-hour capacitated sperm (CS4) are summarized in Table 1. We identified after both capacitation times (CS1 and CS4) a significant decrease in sperm concentration (*p* < 0.05) and an increase in motility and vitality (*p* < 0.05) compared to NCS.

### 3.2. Assessment of Acrosomal Status

**Figure 1:**
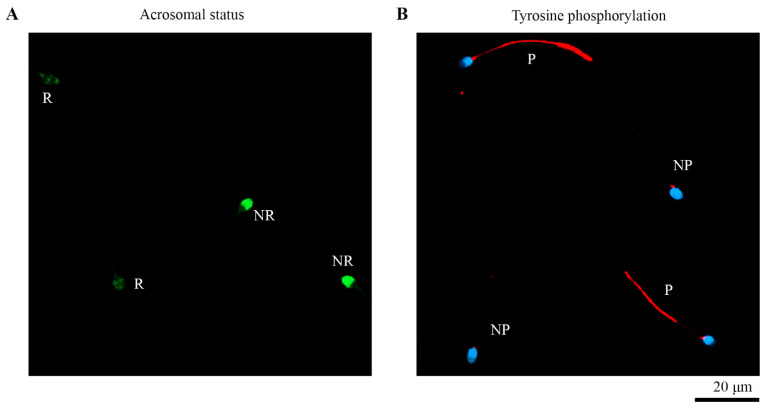
Confocal micrographs of sperm physiological control biomarkers in humans. (**A**) Acrosomal status by PSA-FITC binding label: reacted sperm (R) and non-reacted sperm (NR). (**B**) Tyrosine phosphorylation by PY20 antibody: phosphorylated sperm (P) and non-phosphorylated sperm (NP).

Sperm with label in the acrosome were considered as non-reacted and those with label in the equatorial region were considered as reacted (Figure 1A). Findings showed that in CS1 the percentage of reacted cells was 57.61%, whereas only 22.15% of spontaneous reacted sperm were detected in control (*p* < 0.001; Table 1). Likewise, in CS4, 62.76% of cells indicated positive acrosome reaction, compared to 25.74% from spontaneous acrosome reaction (*p* < 0.001). No variations were noticed in spontaneous and inducted acrosome-reacted cells between both capacitation times (Table 1). However, significant differences (*p* < 0.001) were found after the induction of the acrosome reaction between NCS and capacitated sperm, regardless of the capacitation time (Table 1).

### 3.3. Assessment of Tyrosine Phosphorylation

The phosphorylated state was designated based on the positive label in the flagellum or the absence of it (Figure 1B). Before capacitation, 9.36% of cells were positive for tyrosine phosphorylation; this percentage increased after one-hour capacitation up to 28.41%, and significantly after four-hours’ capacitation up to 37.63% (Table 1). No significant differences in the phosphorylated cell percentage were found between either capacitation times.

### 3.4. SPAM1 Immunolocation

**Figure 2:**
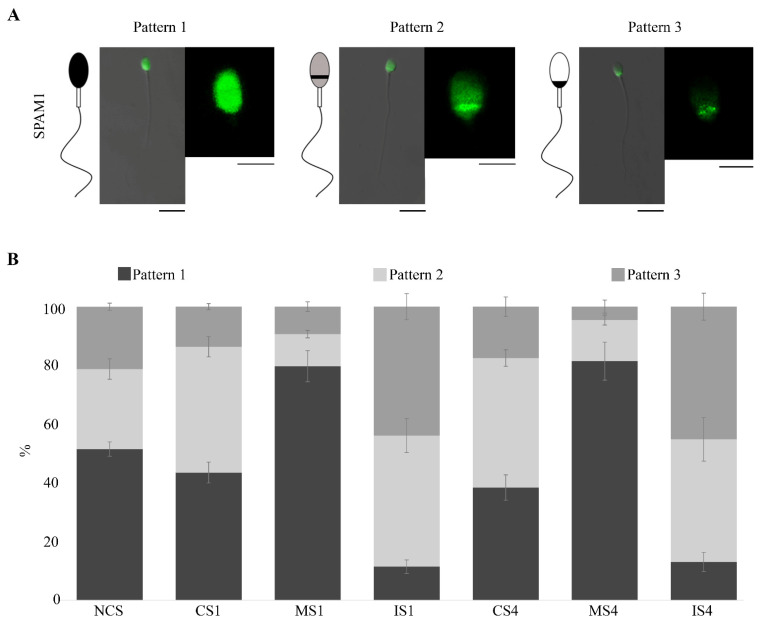
SPAM1 immunolocation results in human sperm. (**A**) Confocal micrographs of sperm SPAM1 patterns. Pattern 1, label throughout the head; pattern 2, equatorial segment with acrosomal faith label; pattern 3, postacrosomal label. Sperm scale 10 µm; head sperm scale 5 µm. (**B**) Percentages and error bars of SPAM1 patterns in each physiological condition. NCS, noncapacitated sperm; CS1, one-hour capacitated sperm; MS1 and IS1, mature and immature sperm selected by hyaluronic acid after one-hour capacitated; CS4, four-hour capacitated sperm; MS4 and IS4, mature and immature selected by hyaluronic acid after four-hour capacitation.

We detected three different SPAM1 fluorescent patterns (Figure 2A): pattern 1 (P1), label throughout the head; pattern 2 (P2), equatorial segment with acrosomal faith label; pattern 3 (P3), postacrosomal label. 

Regarding the location of SPAM1 in each physiological condition, in the NCS condition, we recorded a high subpopulation of cells that presented P1 (~50.00%). Conversely, after both capacitation times (CS1 and CS4), the results reported a significant increase (*p* < 0.05) in the percentage of cells with P2 compared to non-capacitated cells, 42.79% after one hour and 44.08% after four hours’ capacitation (Figure 2B). In addition, no significant differences in SPAM1 localization were reported between different sperm capacitation times. 

Otherwise, despite the increase in P2 during capacitation (CS1 and CS4), the data obtained after selecting the mature sperm by the HA test significantly (*p* < 0.001) highlighted the P1 (label throughout the head) in MS1 and MS4. Specifically, mature sperm selected by HA after one- and four-hour capacitation showed a 79.74 and 81.48% of P1, respectively. This increase in P1 in mature spermatozoa (MS1 and MS4) was significantly different (*p* < 0.001) compared to the rest of the physiological conditions analyzed in this study. However, immature sperm after HA selection in both capacitation times (IS1 and IS4) displayed a high prevalence of P2 (~45.00%) and P3 (~45.00%) and a low presence of P1 (~10.00%). In this case, all the percentages of the different patterns were significantly different (*p* < 0.001) between the immature cells and the mature cells after selection by means of the AH test in both capacitation times (Figure 2B). The statistical data of SPAM1 patterns between the sperm’s different physiological conditions are detailed in the Supplementary Material (Table S1).

## 4. Discussion

Because of high ejaculate heterogeneity [32], sperm selection and capacitation preceding assisted reproduction techniques has been considered an essential point for guaranteeing a positive outcome and an approach to enhance productivity and security [33]. Nevertheless, this guidance is at present weakly investigated, probably due to the absence of fundamental understanding about sperm biomarkers and their heterogeneity [34]. Thus, in this study, we have addressed an exhaustive characterization of SPAM1 location in human sperm. Particularly, we analyzed the localization of this protein before sperm capacitation, at one and four hours of capacitation and after HA selection test. In addition, acrosomal status and tyrosine phosphorylated assays were used as physiological control biomarkers.

In relation to the acrosome reaction, it should be noted that it is an important physiological step for the contact and fusion of the sperm with the oocyte [2,35]. In this study, we analyzed both the spontaneous acrosome reaction and the percentage of acrosome-reacted spermatozoa after in vitro induction. Our results showed spontaneous acrosome reaction rates of around 20%, and rates of 60% after the induction in both spermatozoa capacitation times (CS1 and CS4). In this context, our data complement preceding experiments indicating that the proportion of acrosome-reacted sperm depends on the induction time [36], and the sperm percentage with spontaneous acrosome reaction is around 20% [37]. Moreover, our results showed that sperm capacitation is required for the cells to respond properly to the induction of the acrosome reaction.

Regarding tyrosine phosphorylation, this parameter is commonly used as a sperm capacitation biomarker, since its presence has been linked with sperm hyperactivation, the penetration of cumulus oophorous, and the binding of zona pellucida [38]. In this context, our findings showed that the sperm percentage with tyrosine phosphorylation at the flagellum rose after the in vitro capacitation, noting that the capacitation process was occurring properly. Overall, longer capacitation times positively favored the manifestation of tyrosine phosphorylation, with a significant (*p* < 0.001) percentage of phosphorylated cells after four-hour capacitation. These data are in agreement with preceding research performed in human sperm [39,40] and in other mammalian species [41,42], in which phosphorylation of tyrosine rose in a time-related way [43,44,45,46]. 

The high heterogeneity of both intra- and inter-individual seminal samples gives conventional spermatozoa quality assessment techniques (concentration, motility, morphology, or viability) limited predictive power [47,48]. This great sperm variability may be due, among others, to lifestyle habits, as well as exposure to environmental contaminants such as heavy metals [49] or pollution [50,51]. Therefore, to improve sperm analysis and selection, new potential sperm biomarkers, such as acrosome reaction, protein phosphorylation, DNA damage, and molecules involved in oocyte recognition have been studied [52,53,54]. In this paradigm of new biomarkers, SPAM1 is designated as the main sperm protein with the enzymatic activity necessary to pass through the cumulus layer and reach the oocyte zona pellucida [55]. Furthermore, some authors suggest an important role for SPAM1 in the secondary junctions involved in sperm-zona pellucida binding [7,13,56]. 

To increase knowledge about the localization and redistribution of SPAM1 during sperm capacitation, we have addressed here a comprehensive study of SPAM1 in human sperm. It should be noted that, despite the increase in P2 after sperm capacitation, our results pointed to the coexistence of two major sperm subpopulations in relation to the location of SPAM1. Specifically, in CS1 and CS4 conditions, a high percentage of sperm was obtained for both P1 and P2. These data are complementary to those obtained in previous studies, in which a sperm capacitation of three hours was conducted [5,6]. In general, the coexistence of subpopulations with different SPAM1 patterns underlines the high heterogeneity of semen samples, even in seemingly normozoospermic individuals.

This elevated heterogeneity of the seminal samples presents a challenge in the search for universal fertility biomarkers since the variances are considerably high. In this context, previous research has pointed to COC and HA as potential natural sperm selectors. Specifically, they evaluated the useful role of the COC in sperm selection, and the results indicated that COC-traversing sperm had higher proportions of normal morphology, particular motility patterns, higher zonal binding capacity, and greater chromatin integrity [57,58,59,60]. Furthermore, a previous study suggested that the use of COC to select sperm for ICSI is an efficient procedure, as it significantly improved blastocyst development and quality [23]. Similarly, several studies showed the use of HA favors the selection of mature sperm with adequate DNA integrity, which may optimize ICSI clinical outcome [18,61,62,63], reducing the genetic risk and improving the ICSI treatment safety [21,22]. 

Given the potential of HA as a natural sperm selector, here, we have used this assay after capacitation to further increase selection and recover the sperm subpopulation with the highest reproductive potential. Data obtained after recovering mature sperm by HA test revealed that P1 was by far the most prevalent pattern after both capacitation times (MS1 and MS4). Therefore, as a novelty, our results emphasize that human sperm require the presence of SPAM1 throughout the sperm head (P1) to properly contact the cumulus-oocyte matrix. These results support the usefulness of the HA test as a natural sperm selector [19,29].

Regarding capacitation times used in this study, it is worth mentioning that no significant differences were found in SPAM1 location between one and four hours, neither after capacitation (CS1 and CS4) nor after selection by HA (MS1 and MS4). This suggests that, in the case of the SPAM1 receptor, long sperm capacitation times are not needed for its correct relocation. However, previous studies have highlighted the importance of lengthening the in vitro capacitation time so that sperm can relocate receptors and acquire the ability to recognize the zona pellucida [26,27,64]. This is due to the presence of sperm subpopulations with distinct levels of functionality and membrane cholesterol content [65,66]. Therefore, each sperm receptor requires a different in vitro capacitation time for its relocation. 

## 5. Conclusions

In conclusion, our results provide novel insights into the SPAM1 protein physiological basis during sperm capacitation and HA test selection, which could contribute to the improvement of sperm selection techniques for assisted reproductive technologies. Overall, due to the high heterogeneity of the human sperm, future research should focus on selecting a subpopulation with greater reproductive potential. 

## Figures and Tables

**Table 1:** Semen samples parameters.

Parameter	NCS Mean ± SD	CS1 Mean ± SD	CS4 Mean ± SD
Volume (mL)	3.16 ± 0.97	-	-
pH	7.28 ± 0.96	-	-
Normal morphology (%)	13.35 ± 3.99	-	-
Concentration (10^6^/mL)	62.93 ± 22.29	19.00 ± 18.42 *	11.05 ± 7.96 *
Total motility (%)	73.95 ± 10.26	97.54 ± 1.26 *	96.48 ± 1.78 *
Viability (%)	80.87 ± 8.09	98.75 ± 3.15 *	96.22 ± 2.67 *
Spontaneous acrosome reaction (%)	19.24 ± 5.54	22.15 ± 7.18	25.74 ± 6.47
Induced acrosome reaction (%)	37.95 ± 10.88	57.61 ± 12.36 *	62.76 ± 15.76 *
Tyrosine phosphorylation (%)	9.36 ± 4.52	28.41 ± 8.27	37.63 ± 10.32 *

Noncapacitated sperm (NCS), one-hour capacitated sperm (CS1), four-hour capacitated sperm (CS4). * Kruskal–Wallis test *p* < 0.001 to NC.

## Data Availability

Not applicable.

## Supplementary Materials

The following supporting information can be downloaded at: https://www.mdpi.com/article/10.3390/biomedicines10102553/s1, Table S1: Statistical data of SPAM1 patterns between different sperm physiological conditions.
