# Supplementary material for: Sperm Adhesion Molecule 1 (SPAM1) Distribution in Selected Human Sperm by Hyaluronic Acid Test
Source: Biomedicines. 2022 Oct 13;10(10):2553. doi: 10.3390/biomedicines10102553 (PMC9599839; doi:10.3390/biomedicines10102553)
Supplement: Supplementary file 1 [file biomedicines-10-02553-s001.zip › biomedicines-1955701-supplementary.pdf]

Table S1. Statistical data of SPAM1 patterns between different sperm physiological conditions.

Significance between Physiological Conditions							Patterns	Physiological Condition
**NCS**	**CS1**	**MS1**	**IS1**	**CS4**	**MS4**	**IS4**		
-	n.s.	**	**	n.s.	**	**	P1	NCS
-	*	**	n.s.	*	**	n.s.	P2	NCS
-	n.s.	n.s.	**	n.s.	*	**	P3	NCS
	-	**	**	n.s.	**	**	P1	CS1
	-	**	n.s.	n.s.	**	n.s.	P2	CS1
	-	n.s.	**	n.s.	n.s.	**	P3	CS1
		-	**	**	n.s.	**	P1	MS1
		-	**	**	n.s.	**	P2	MS1
		-	**	*	n.s.	**	P3	MS1
			-	**	**	n.s.	P1	IS1
			-	n.s.	**	n.s.	P2	IS1
			-	**	**	n.s.	P3	IS1
				-	**	**	P1	CS4
				-	**	n.s.	P2	CS4
				-	*	**	P3	CS4
					-	**	P1	MS4
					-	**	P2	MS4
					-	**	P3	MS4
						-	P1	IS4
						-	P2	IS4
						-	P3	IS4

NCS, noncapacitated sperm; CS1, one-hour capacitated sperm; MS1 and IS1, mature and immature sperm selected by hyaluronic acid after one-hour capacitated; CS4, four-hour capacitated sperm; MS4 and IS4, mature and immature selected by hyaluronic acid after four-hour capacitated.

** Kruskal-Wallis test $p < 0.001$; * Kruskal-Wallis test $p < 0.05$; n.s. not significant.
